# Global trends and hotspots of phage therapy for bacterial infection: A bibliometric visualized analysis from 2001 to 2021

**DOI:** 10.3389/fmicb.2022.1067803

**Published:** 2023-01-09

**Authors:** Zulipikaer Maimaiti, Zhuo Li, Chi Xu, Jiying Chen, Wei Chai

**Affiliations:** ^1^Department of Orthopedics, The Fourth Medical Center, Chinese PLA General Hospital, Beijing, China; ^2^Department of Orthopedics, The First Medical Center, Chinese PLA General Hospital, Beijing, China; ^3^School of Medicine, Nankai University, Tianjin, China

**Keywords:** phage therapy, bacterial infection, hotspot, research trends, bibliometrics

## Abstract

**Background:**

Antibiotic resistance is one of the main global threats to human health, and just the development of new antimicrobial medications is not enough to solve the crisis. Phage therapy (PT), a safe and effective treatment method, has reignited the interest of researchers due to its efficacy in the clinical treatment of drug-resistant bacterial infections. There is, however, no bibliometric analysis of the overall trends on this topic. Therefore, this study aims to provide an overview of the current state of development and research in this area.

**Methods:**

We extracted all relevant publications from the Web of Science Core Collection (WoSCC) database between 2001 and 2021. We performed bibliometric analysis and visualization using CiteSpace, VOS viewer, and R software. Annual trends of publications, countries/regions distributions, institutions, funding agencies, co-cited journals, author contributions, core journals, references, and keywords were analyzed.

**Results:**

A total of 6,538 papers were enrolled in this study, including 5,364 articles and 1,174 reviews. Publications have increased drastically from 61 in 2001 to 937 in 2021, with 3,659 articles published in the last 5 years. North America, Western Europe, and East Asia were significant contributor regions. The United States, China, and the United Kingdom were the most productive countries. The Polish Academy of Sciences was the most contributive institution. *Frontiers in Microbiology* and *Applied and Environmental Microbiology* were the most productive and co-cited journals. A. Gorski and R. Lavigne published most articles in this field, while V. A. Fischetti was the author with the most cited. Regarding keywords, research focuses include phage biology, phage against clinically important pathogens, phage lysis proteins, phage therapy, biofilm-related research, and recent clinical applications.

**Conclusion:**

Phage therapy is a potential strategy for combating antibiotic resistance, and it will provide us with an alternative therapeutic option for bacterial infection. According to global trends, the scientific output of PT in bacterial infections is increasing, with developed countries such as the United States leading the way in this area. Although the safety and efficacy of PT have been proven, more clinical trials on the phages against infectious diseases caused by various pathogens are still needed.

## Introduction

Antibiotic resistance has become a serious threat to human health. The increasing quantity and spread of antibiotic-resistant strains confer a substantial burden on healthcare systems worldwide, and one study predicts that antibiotic-resistant diseases will kill around 10 million people worldwide by 2050 (Willyard, 2017). According to a 2013 Centers for Disease Control and Prevention (CDC) report, at least 2 million people are infected with antibiotic-resistant bacteria each year, and about 23,000 people die (Septimus and Schweizer, 2016). The development of novel antibiotics is characterized by long cycles, high costs, and high risks, so there is an urgent need to explore new adjuvant antimicrobial treatment approaches. In this context, phage therapy (PT) has received renewed attention. PT is well qualified as a complementary approach to combat antibiotic resistance, and numerous studies conducted over the past few years have demonstrated PT’s novel potential.

Phages (also known as bacteriophages) are bacteria-infecting viruses that were formally discovered more than a century ago by microbiologist Frederik Twort, termed “bacteriophage” in 1917 by Félix d’Hérelle, which means “bacterium eater” (Chanishvili, 2012). Phages are common in the environment and probably the most ancient and ubiquitous viruses on earth, with a number around 10 times that of their host bacteria (Burrowes et al., 2011). Phages are typically 20–400 nm in size and consist of DNA or RNA, protein capsids, and tail fibers that engage with various bacterial surface receptors (Letchumanan et al., 2016; Criscuolo et al., 2017). Many properties distinguish phages, including life cycle, nucleic acid concentration, and viral particle form (Young, 1992). Phages can be classified into two classes based on their life cycle: lytic phages and temperate phages. Lysis phages cause lysis of the bacterial host through a form of infection and create the next generation of phage progeny, which will continue to infect the surrounding environment (Young, 1992). However, temperate phages can alter the properties of the bacterium without causing severe harm to the host cell by inserting their genomes into the host chromosome during lysogeny and then remaining dormant in the bacterial cell (Kahn et al., 2019). As a result, lytic phages are more ideal for use in biotherapeutics (Sillankorva et al., 2012). Though Hérelle used phages to treat bacterial infections in humans for the first time, after Alexander Fleming discovered penicillin in 1928, Western medicine began widely using antibiotics in the 1940s (Ho, 2001; Alcock et al., 2020). In some ways, PT has taken a back seat to the development of antibiotics. Even so, some scientists continued studying PT, which has steadily grown in places like Georgia (part of the former Soviet Union) and Poland.

Phages have been used to treat human bacterial infections for almost 100 years. Since the 21st century, more and more studies have let people better understand phage biology and immunology, and the safety of PT is guaranteed with the use of many modern technologies, such as whole-genome sequencing and automation (Kortright et al., 2019). PT is currently being utilized to treat pneumonia, gastrointestinal infections, peritonitis, dysentery, typhoid, urinary tract infections, burns, and implant-associated infections with success (Chen et al., 2019; Gordillo Altamirano and Barr, 2019; Cano et al., 2020). The FDA approved a Phase I clinical trial in 2006 to investigate the safety of phage-based therapies for difficult-to-treat wounds, and this study indicated no safety concerns with this phage treatment (Rhoads et al., 2009). In 2009, a clinical investigation in Bangladesh assessed the efficacy of oral T4 phage in treating acute bacterial diarrhea in young children; although this study did not show clinical efficacy, it also demonstrated the safety of phage therapy (Sarker et al., 2016). Another clinical trial reported in 2009 revealed that the therapeutic phage formulation considerably reduced the antibiotic-resistant *Pseudomonas aeruginosa* burden without causing any adverse events, proving that PT is efficacious and safe in chronic otitis media (Wright et al., 2009).

Many promising clinical trials and case reports of personalized phage therapies have been published recently. In 2015, a randomized, multicenter, open-label phase I/II clinical trial was conducted in Europe to assess the efficacy of a cocktail of anti-*Pseudomonas aeruginosa* phages for treating patients with burn wound infections. Natural lytic anti-*P aeruginosa* bacteriophages decreased bacterial burden in burn wounds at a slower rate than standard therapy, but patients did receive the clinical benefit in this treatment protocol (Jault et al., 2019). In single-arm non-comparative research conducted in Australia in 2017, 13 patients with severe *S*. *aureus* infections were given three Myoviridae bacteriophages (AB-SA01), and the study found that PT was safe and effective in treating severe *S*. *aureus* infections (Petrovic Fabijan et al., 2020). Another randomized, double-blind, placebo-controlled crossover trial conducted in the United States in 2018, which included 32 patients taking capsules containing four phages targeting known gastrointestinal pathogens, demonstrated that PT was safe and well tolerated in people with mild to moderate gastrointestinal distress (Gindin et al., 2019). Schooley et al. (2017) reported the first successful case of *Acinetobacter baumannii* infection treatment with phage in the United States, and Dedrick et al. (2019) reported the first treatment of *Mycobacterium* infections with designed phage. According to Chan et al. (2018), *Pseudomonas aeruginosa* infected aortic grafts were also successfully treated with phage. Cano et al. (2020) first employed phages in 2021 to treat infections in patients with periprosthetic joint infection (PJI) with satisfactory results, providing new hope in cases of difficult-to-treat biofilm-associated prosthetic joint infections. PT has a bright future in individualized treatment and will also be a novel tool for combating the ongoing challenge of antibiotic resistance in modern clinical treatment. Consequently, PT for bacterial infections has received renewed and widespread attention worldwide.

The rapid accumulation of knowledge in the field makes it hard for researchers to comprehend it well. Bibliometric analysis is a statistical and analytical procedure used to summarize the current state of research, identify research frontiers and hotspots, and further predict future trends (Chen and Song, 2019; Ma et al., 2022). Besides, bibliometric analysis is frequently integrated with visual data to examine and compare the contributions of various countries, institutions, journals, and researchers, as well as their relationships (Zhang et al., 2022). To the best of our knowledge, there are no bibliometric studies that offer a thorough and integrated evaluation of PT in the management of bacterial infections. In this paper, we use bibliometric analysis to describe the research on PT for bacterial infections over the past 20 years, figure out what it is about, and try to predict future research trends and hotspots to help guide future research.

## Materials and methods

### Data source and search strategies

**Figure 1:**
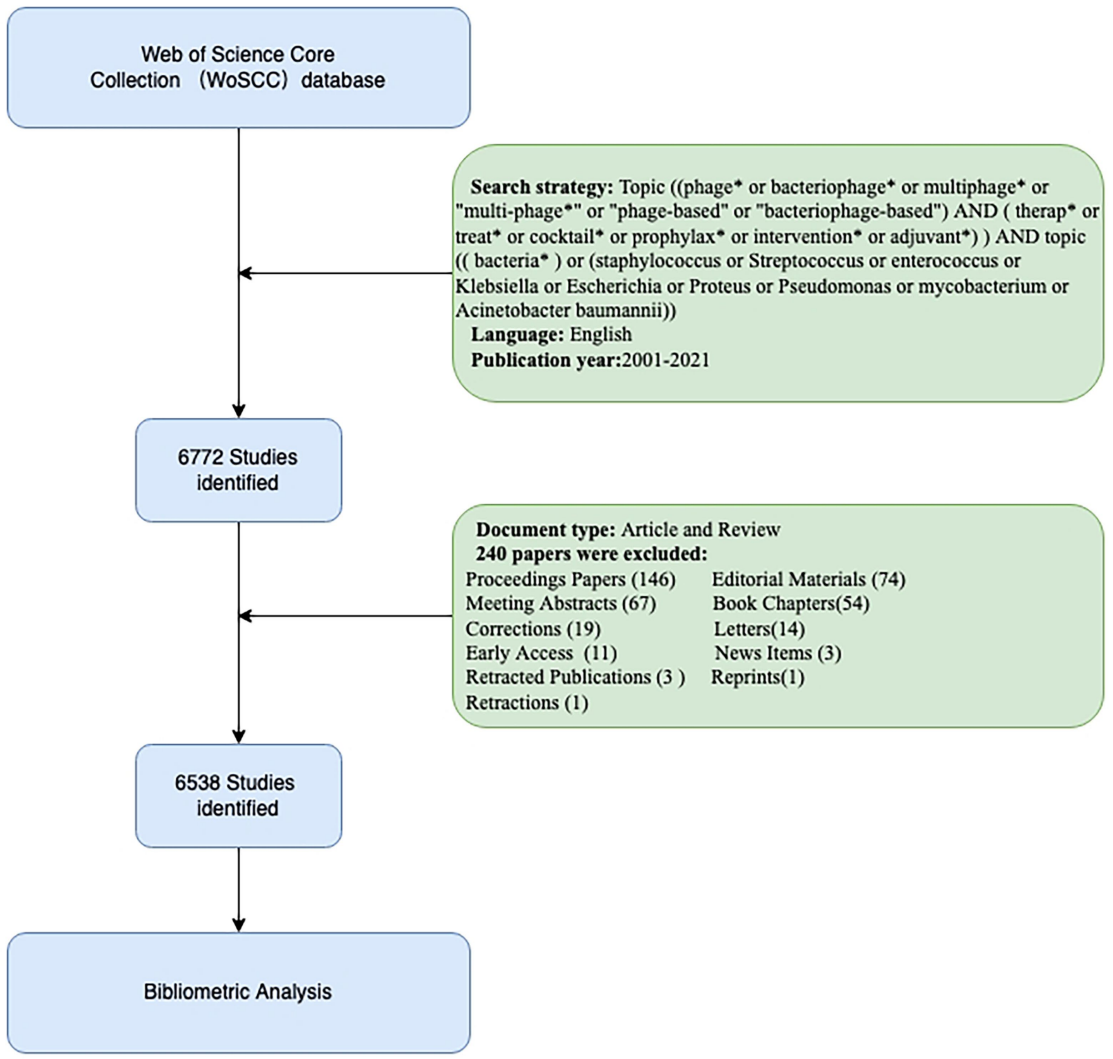
Flowchart of the data screening process.

The Web of Science Core Collection (WoSCC) database from Clarivate Analytics is widely used for bibliometric analysis. In our study, the WoSCC database was searched for articles published between 2001 and 2021. The following search strategies were presented: topic[(phage* or bacteriophage* or multiphage* or “multi-phage*” or “phage-based” or “bacteriophage-based”) AND (therap* or treat* or cocktail* or prophylax* or intervention* or adjuvant*)] AND [(bacteria*) or (*Staphylococcus* or *Streptococcus* or *Enterococcus* or *Klebsiella* or *Escherichia* or *Proteus* or *Pseudomonas* or *Mycobacterium* or *Acinetobacter baumannii*)]. Our analyses were conducted only on English-language reviews and articles. All of the papers retrieved from the WoSCC were downloaded as “Full Record and Cited References” and saved as “plain text.” In the end, 6,538 documents were analyzed in our study. The enrollment and screening procedures are described in Figure 1. To avoid bias caused by database updates, the above data were obtained from the WoSCC database on June 27, 2022.

### Data extraction

Total records were downloaded from the WoSCC database, including journals, publication dates, article titles, authors, institutions, original countries or regions, abstracts, keywords, the publication’s source, funding agencies, H-index, and citation frequency. Then, all the documents were imported to Microsoft Excel 2019, CiteSpace V, GraphPad Prism 6, VOS viewer 1.6.18, or R for further bibliometric analysis and data visualization. The H-index is defined as the number of h papers published, each of which has been cited at least h times. It is not only used to evaluate individual scholarly achievements but can also describe the publication output of a country or region, an institution, or a journal and is superior to total publication and citation counts in predicting future scientific productivity (Jones et al., 2011). The latest version of the journal citation report (JCR) was used to obtain the impact factor (IF), quartile of a journal category (Q1, Q2, Q3, and Q4) for evaluating the journal’s performance within its field. To ensure the authenticity of the data, two qualified authors (ZM and ZL) independently screened and extracted key information from the final included articles and then analyzed the data together. Any disagreement during the review of the complete text will be resolved through negotiation.

### Bibliometric and visualized analysis

Microsoft Office Excel was used to analyze the trends of annual publications and the citations of the included literature. The Bibliometrix and Biblioshiny packages in R were used to conduct collaboration network analysis among countries. We used an online platform to analyze each country’s annual volume of articles.^1^ Country/organization distributions, author contributions, core journals, keyword co-occurrences with co-authorship, co-citation, and co-occurrence analyses in default settings, and display visualizations of network maps of these items performed by VOS viewer software (van Eck and Waltman, 2010). Total link strength (TLS) reflects link strength between indicators quantitatively. Moreover, we also performed the co-citation analysis in co-cited references using CiteSpace software (Chen et al., 2014). The settings were: timespan (2001–2021), with years per slice one. The selection uses a modified g-index in each slice: $g^{2}\leq \underset{i\leq g}{\sum }c_{i},k\epsilon z^{+}$ , the scale factor *k* = 25 and shows cluster labels by light semantic indexing (LSI). The minimum duration for burstiness in co-cited references was set to 4 years with the default settings. The strongest citation bursts indicate that the study was cited extensively within a certain period. Citation bursts are often used in bibliometrics to show the most frequently cited references in a specific period, which helps to understand the frontiers of this research field better. Hence, using CiteSpace software, we also analyzed the top 25 references with the strongest citation bursts from 2001 to 2021.

### Research ethics

The data came from public databases, and there were no human or animal subjects. This bibliometric analysis does not require ethics committee approval.

## Results

### An overview of annual growth trend

**Figure 2:**
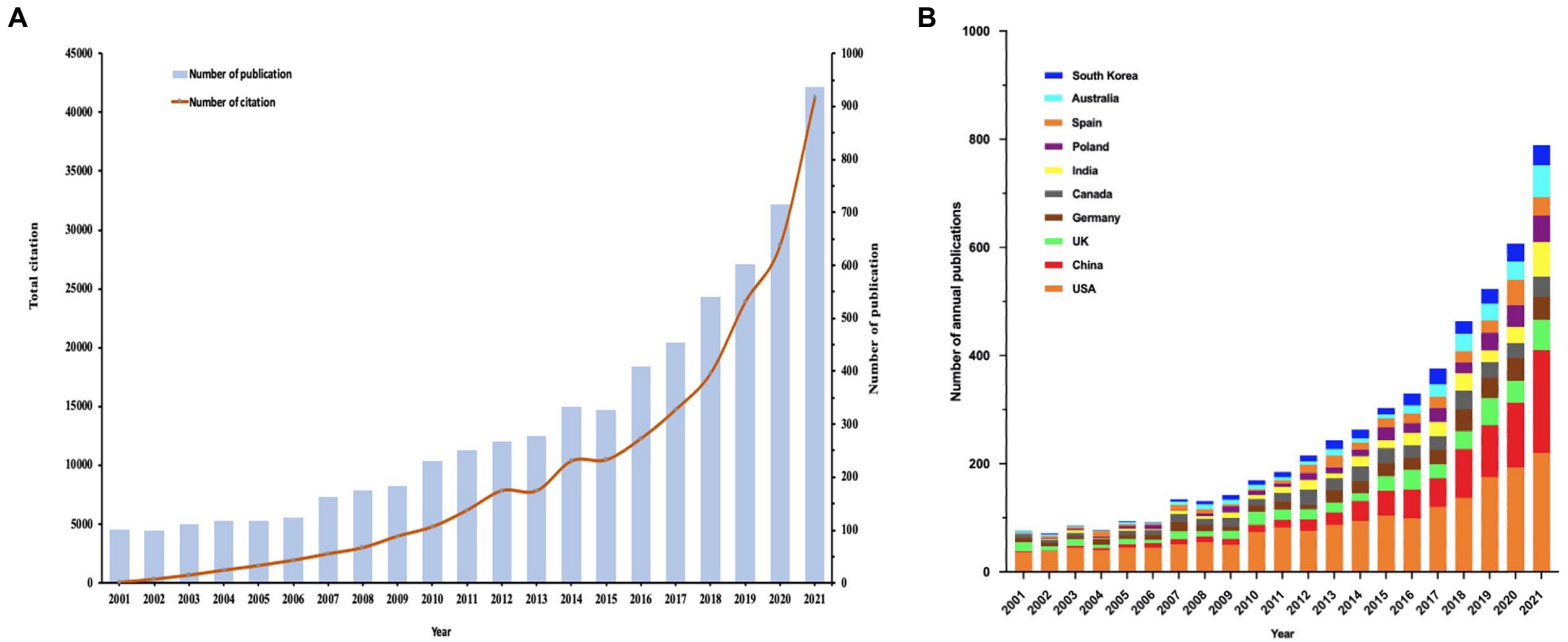
**(A)** Global trend of annual publications and citations on PT from 2001 to 2021. **(B)** The country’s annual trend publications.

We obtained a total of 6,772 initial records from the WoSCC database, and finally, 6,538 publications that met the inclusion criteria were enrolled in this study, including 5,364 articles and 1,174 reviews (Figure 1). The annual number of publications and the total number of citations (Figure 2A) presented that the number of global PT studies on the bacterial infection has steadily increased in the past 20 years. According to our statistics, publications have increased drastically from 61 in 2001 to 937 in 2021, with 3,659 articles published in the last 5 years accounting for 56% of all publications. All of these publications have been cited 227,633 times, and each paper has been cited 35 times on average. The annual publication number of the top 10 countries from 2001 to 2021 is illustrated in Figure 2B. Since 2001, developed countries such as the United States and the United Kingdom have been the most dominant output countries, but this trend has gradually changed over the last 5 years as China and India have been contributing more and more to the field; in 2021, China was second only to the United States in terms of annual publications.

### Contributions of countries/regions

**Figure 3:**
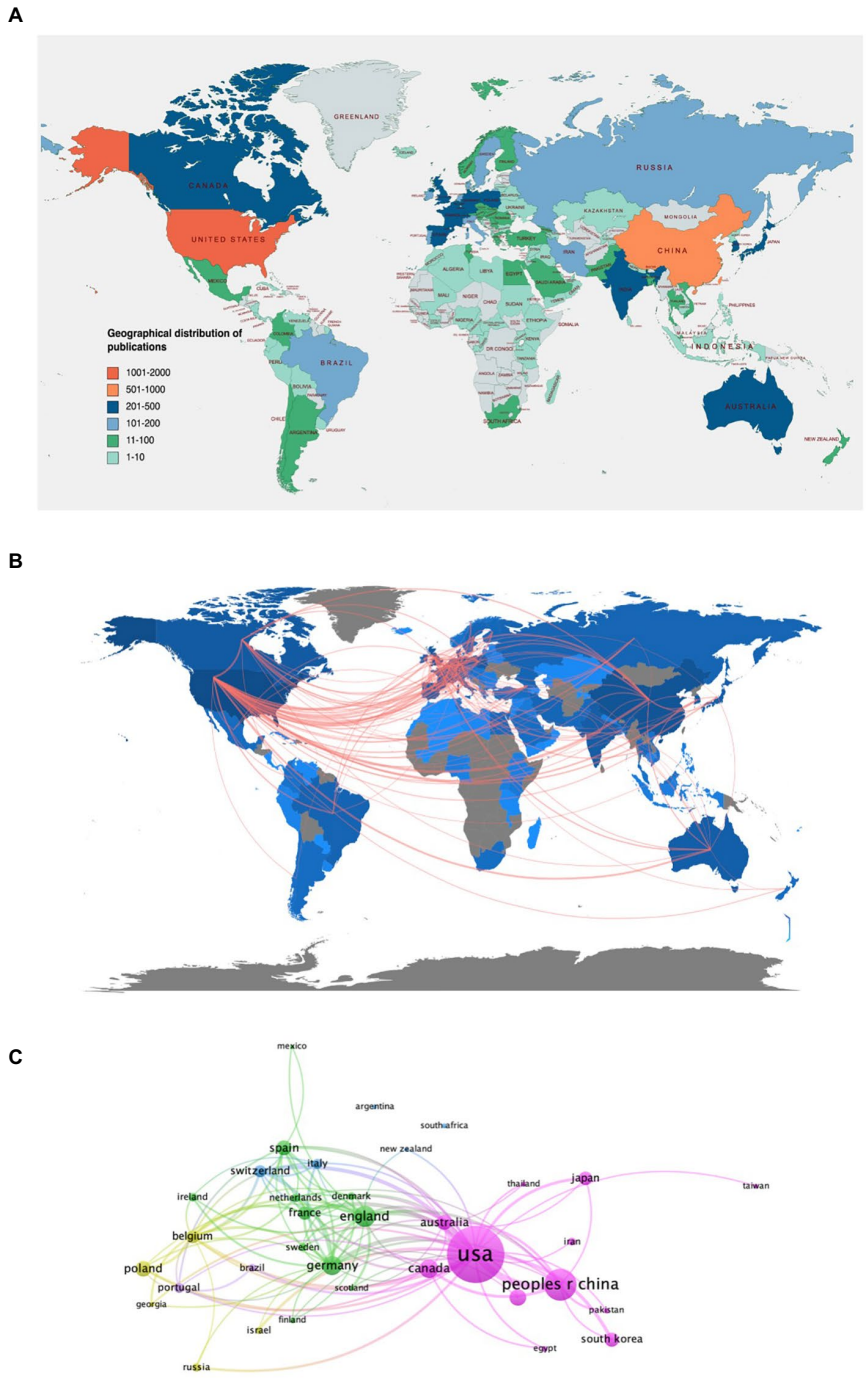
**(A)** World map of the global distribution. **(B)** World map of the global collaborations among countries in the field of PT therapy on bacterial infection. The thicker the red line, the more frequent the collaboration. **(C)** Collaboration networks among countries or regions based on VOS viewer.

One hundred nineteen countries or regions contributed publications related to this topic. The world map shows the total number of publications from different countries or regions. As shown in Figure 3A, the different colors represent different thresholds. The geographical distribution of global publications revealed that studies of PT were published all over the world, mainly in North America, Western Europe, and East Asia. At the same time, the majority of countries in Africa, Western Asia, and South America rarely contributed to this field. Table 1 listed the most productive countries, and the top five countries were the United States (1,867 publications, 28.56%), China (811 publications, 12.41%), the United Kingdom (463 publications, 7.08%), Germany (407 publications, 6.23%), and Canada (381 publications, 5.83%). Regarding the H-index, the United States is the highest and almost twice as high as the United Kingdom, Canada, and Germany. Furthermore, publications from the United States have 4–5 times the total number of citations compared to China, Canada, the UK, and Germany.

**Table 1:** The top 10 productive countries/regions related to phage therapy in bacterial infection.

Rank	Country	No. of articles	Total citation	% of (6,538)	Citation per publication	H-index	TLS
1	United States	1,867	84,811	28.56	45.43	135	861
2	China	811	14,648	12.41	18.06	51	341
3	United Kingdom	463	19,043	7.08	41.13	72	412
4	Germany	407	15,219	6.23	37.39	65	385
5	Canada	381	15,097	5.83	39.62	67	295
6	India	310	7,012	4.74	22.62	46	81
7	Poland	296	8,112	4.53	27.41	50	149
8	Spain	285	9,649	4.36	33.86	55	234
9	Australia	279	9,529	4.27	34.15	53	248
10	South Korea	271	5,839	4.15	21.55	40	72

The international collaboration network (Figure 3B) and country collaboration map (Figure 3C) demonstrated close cooperation among countries or regions. As the largest contributor, the United States collaborates closely with Canada, Australia, China, and European countries, mainly the United Kingdom, Germany, and France. As the second largest contributing country, China actively cooperates with several European countries and closely cooperates with the United States, South Korea, and Japan.

### Contributions of institutions

**Figure 4:**
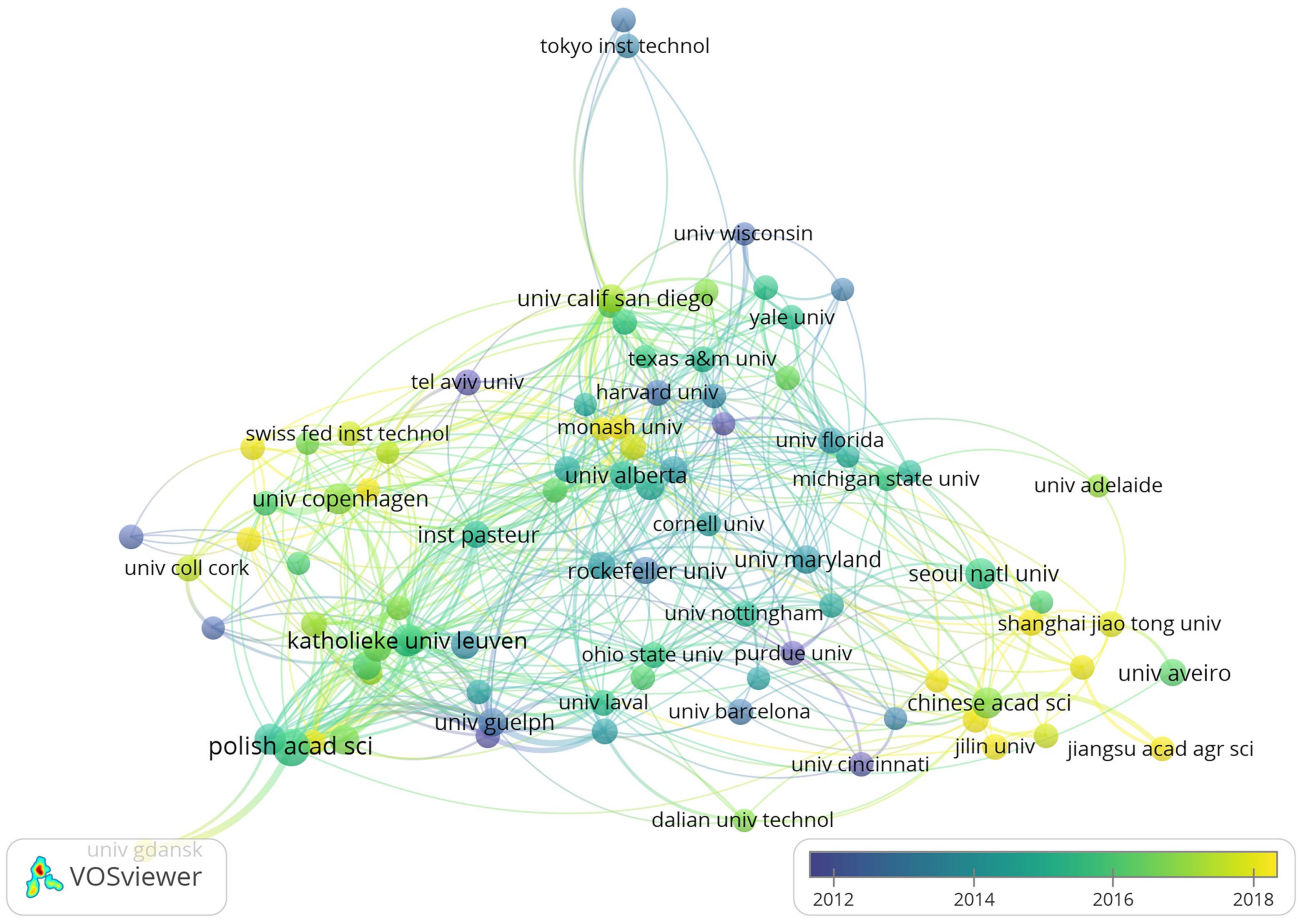
A visual map of institutional collaboration analysis.

The top 10 most productive institutions are listed in Table 2. Most of them were from European countries. The Polish Academy of Sciences is the most productive institution (192 publications, 5,721 citations), followed by Ku Leuven University (112 publications, 5,532 citations) and the Chinese Academy of Sciences (103 publications, 2,015 citations). Based on the H-index, the top three institutions were Ku Leuven University, the Polish Academy of Sciences, and Harvard University. Harvard University had the highest average number of citations per publication, followed by Ku Leuven University and Ghent University. Institutional cooperation visualization analysis was generated by the VOS viewer, which was produced to reveal the cooperation between institutions (Figure 4). The purple means that the institution published the study fairly early, while the yellow means that the institution published the study more recently.

**Table 2:** Top 10 related affiliations.

Rank		Country	No. of articles	Total citation	% of (6,538)	Citation per publication	H-index
1	Polish Academy Of Sciences	Poland	192	5,721	2.937	29.80	45
2	Ku Leuven University	Belgium	112	5,532	1.713	49.39	46
3	Chinese Academy Of Sciences	China	103	2015	1.575	19.56	26
4	Seoul National University	South Korea	88	2,440	1.346	27.73	30
5	University Of Copenhagen	Dan mark	83	2,708	1.270	32.63	27
6	Harvard University	United States	82	5,597	1.254	68.26	37
7	Medical University Of Warsaw	Poland	82	2,933	1.254	35.77	30
8	Russian Academy Of Sciences	Russia	80	1,613	1.224	20.16	23
9	Ghent University	Netherland	79	3,728	1.208	47.19	34
10	Universidade Do Minho	Portugal	78	3,309	1.193	42.42	32

### Contributions of funding agencies

The top 10 funding agencies for research on the PT of bacterial infections are listed in Table 3. All funding agencies are from North America and Europe, except for China. The United States has six funding agencies as major contributors, with the United States Department of Health and Human Services (HHS) and the United States National Institutes of Health (NIH) in first and second place, respectively. Although China has only one funding agency, the National Science Foundation of China (NSFC) ranks third.

**Table 3:** Top 10 related funding agencies.

Rank	Funding agencies	Countries/Regions	Count	Percentage% (*N*/6088)
1	United States Department of Health Human Services (HHS)	United States	683	10.447
2	National Institutes of Health (NIH)	United States	670	10.248
3	National Natural Science Foundation of China (NSFC)	China	443	6.776
4	European Commission	EU	423	6.47
5	NIH National Institute of Allergy Infectious Diseases (NIAID)	United States	252	3.854
6	United Kingdom Research Innovation (UKRI)	United Kingdom	204	3.12
7	NIH National Institute of General Medical Sciences (NIGMS)	United States	203	3.105
8	National Science Foundation (NSF)	United States	142	2.172
9	Biotechnology and Biological Sciences Research Council (BBSRC)	United Kingdom	125	1.912
10	Natural Sciences and Engineering Research Council of Canada (NSERC)	Canada	116	1.774

### Analysis of journals and co-cited journals

**Figure 5:**
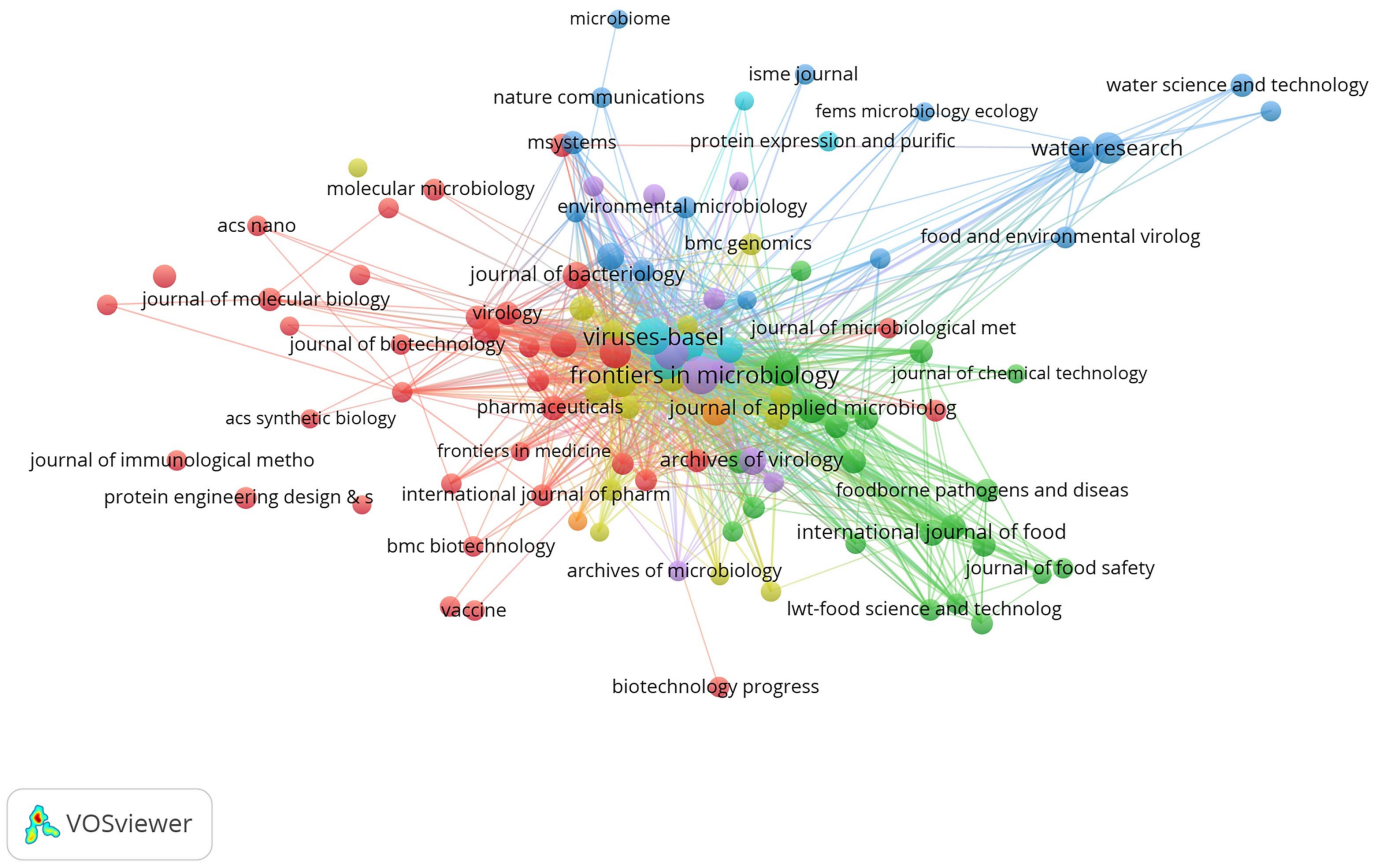
The network visualization of journals co-citation relationship.

As shown in Table 4, the top 10 most productive journals published 1,572 publications, accounting for 24.04% of all publications. Three of the top 10 most productive journals were classified as Q1 and five as Q2, with two journals classified as Q1 and Q2 according to different disciplines. *Frontiers in Microbiology* (IF = 6.064) published the most articles/reviews (297 publications), followed by *Viruses Basel* (207 publications, IF = 5.818) and *Applied and Environmental Microbiology* (203 publications, IF = 5.005). According to the 2021 JCR report, the IF of the top 10 most productive journals ranged from 3.752 to 13.4. The top three highest-cited journals were *Applied and Environmental Microbiology* (citations = 9,710), *Antimicrobial Agents and Chemotherapy* (citations = 8,425), and *Frontiers in Microbiology* (citations = 8,275). The co-citation relationship among different journals was visualized in a co-citation network (Figure 5). These journals also interact with each other. *Frontiers in Microbiology* and *Viruses Basel* are at the center and have close cooperation.

**Table 4:** The top 10 productive journals related to phage therapy in bacterial infection.

Ranking	Journal title	Count	Total citation	Percentage (*N*/6,538)	Citation per publication	IF (2021)	Quartile in category (2021)
1	Frontiers in Microbiology	297	8,275	4.543	27.86	6.064	Q1
2	Viruses Basel	207	4,166	3.166	20.13	5.818	Q2
3	Applied and Environmental Microbiology	203	9,710	3.105	47.83	5.005	Q2
4	Plos One	182	6,858	2.784	37.68	3.752	Q2
5	Antibiotics Basel	132	1,579	2.019	11.96	5.222	Q1/Q2
6	Scientific Reports	120	2,938	1.835	24.48	4.996	Q2
7	Antimicrobial Agents and Chemotherapy	113	8,425	1.728	74.56	5.938	Q1/Q2
8	Water Research	97	6,356	1.484	65.53	13.4	Q1
9	Journal of Applied Microbiology	87	3,273	1.331	37.62	4.059	Q2
10	Applied Microbiology and Biotechnology	74	2,735	1.132	36.96	5.56	Q1

### Contributions of authors

**Figure 6:**
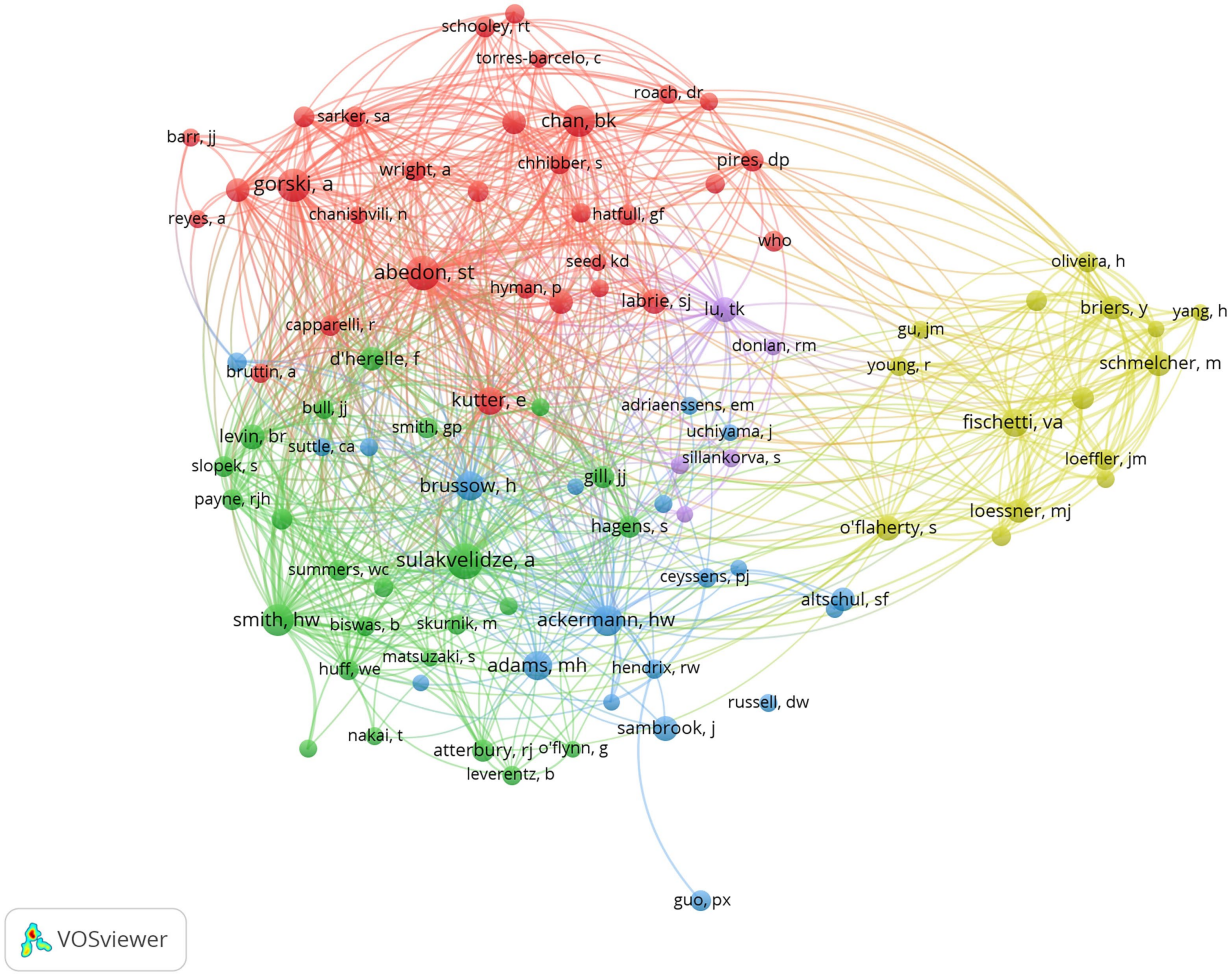
Top 100 most productive authors in the field (T ≥ 200).

The 10 most productive authors are listed in Table 5. Most of the authors were European, with four from Poland, two from Spain, one from Belgium, and one from Portugal. The United States and Australia had one author each. A. Gorski from the Polish Academy of Sciences contributed the most publications (104), followed by R. Lavigne (82) from Ku Leuven University and B. Weber-Dabrowska (71) from the Polish Academy of Sciences. To better visualize the co-cited authors, co-citations are set at 200 and get 96 authors, and the results are shown in Figure 6. The top three co-cited authors were S. T. Abedon, A. Sulakvelidze, and A. Gorski, which is notable.

**Table 5:** Top 10 authors related to phage therapy in bacterial infection.

Ranking	Label	Country	No. of articles	Total citation	% of (6,538)	Citation per publication	H-index
1	A. Gorski	Poland	104	3,909	1.591	37.59	35
2	R. Lavigne	Belgium	82	3,729	1.254	45.48	37
3	B. Weber-Dabrowska	Poland	71	2,893	1.086	40.75	30
4	P. Garcia	Spain	59	2,295	0.902	38.9	29
5	J. Azeredo	Portugal	54	2,552	0.826	47.26	29
6	K. Dabrowska	Poland	46	1985	0.704	43.15	23
7	R. Miedzybrodzki	Poland	45	1709	0.688	37.98	23
8	V.A. Fischetti	United States	42	4,315	0.642	102.74	31
9	A. Rodriguez	Spain	42	1,636	0.642	38.95	24
10	S. Morales	Australia	41	1,360	0.627	33.17	21

### Analysis of cited references

**Figure 7:**
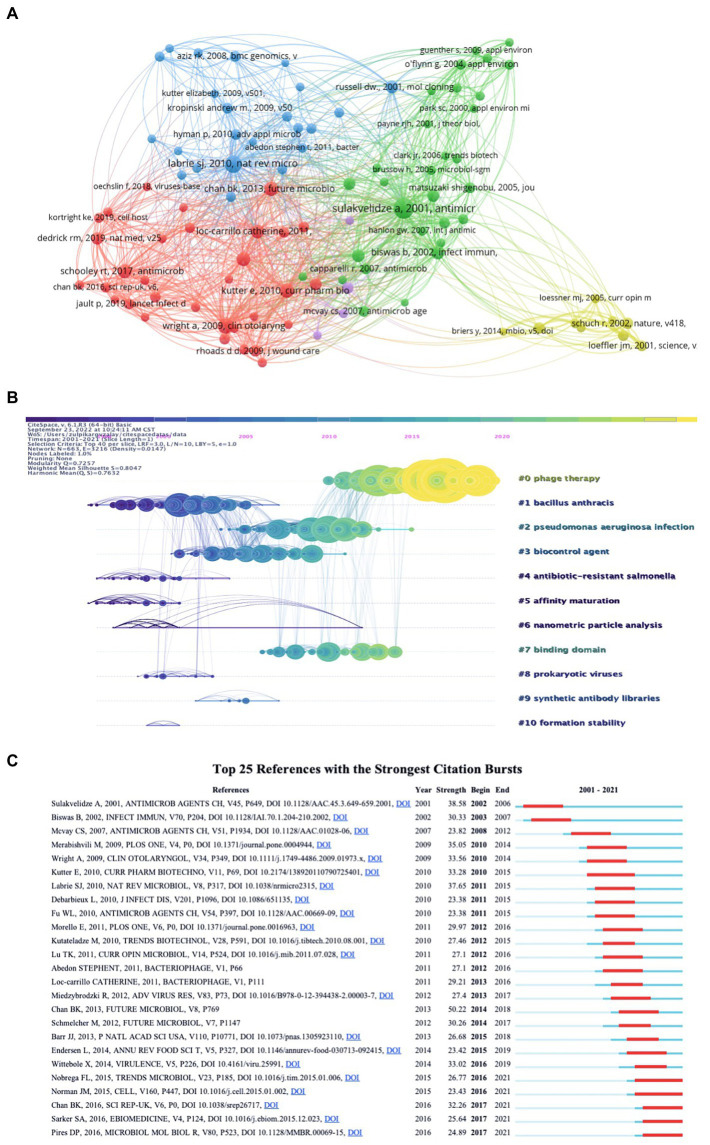
Visualization mapping of co-cited references. **(A)** Network map of co-cited references. **(B)** Cluster view, top 25 references with strongest citation bursts based on Cite Space. **(C)** Timeline view, the position of the nodes on the horizontal axis indicates the time when the reference first appeared, the 10 clusters are marked and color coded on the right side, the nodes are large and dense indicating which was the hot topics in that time period, the time evolution is indicated by different colored lines, the more yellow the color indicates closer to 2021.

The top 15 co-cited references related to PT for bacterial infection are listed in Table 6. All the top 15 references were co-cited at least 342 times. Highly cited publications were mainly published between 2001 and 2010, with 14 studies and four published after 2010. The most highly cited paper was written by Weinbauer (2004) with 1,096 citations and published in *FEMS Microbiology Reviews* (Weinbauer, 2004). The second-most-cited article was published by Sulakvelidze et al. (2001) in *Antimicrob Agents Chemother*, with 973 citations (Sulakvelidze et al., 2001). The paper written by Lu and Collins (2007) ranked third and was published in *The Proceedings of the National Academy of Sciences* (*PNAS*) with 516 citations (Lu and Collins, 2007). To better visualize the co-cited references network, we selected 98 authors with at least 100 co-citations and analyzed them by VOS Viewer, and the results are shown in Figure 7A. This network map showed that Sulakvelidze, Loc-Carrillo Catherine, and Schooley et al. were in a central position. Figure 7B shows that the blue bars represent the time interval, and the red bars represent the citation burst of references. The first citation burst of references emerged in 2002, and the study was published by A. Sulakvelidze in 2001 (Sulakvelidze et al., 2001). Twenty articles or reviews were published after 2010, with most published between 2010 and 2011. The most recent reference with the strongest citation burst was seen in 2015, and the citation burst kept going. Moreover, we performed a temporal co-citation analysis and plotted the timeline view of co-cited references (Figure 7C). The results indicated that #5 (affinity maturation) and #1 (Bacillus anthracis) were relatively early hotspots, #2 (*Pseudomonas aeruginosa* infection), #3 (biocontrol agent), and #7 (binding domain) were mid-term (2005–2015) research hotspots and #0 (phage therapy) was the most recent term (2010–2021).

**Table 6:** Top 15 co-cited references related to phage therapy on bacterial infection.

Ranking	Title	Total citations	First author	Year	Journal
1	Ecology of prokaryotic viruses	1,096	M. G. Weinbauer	2004	Fems. Microbiol. Rev.
2	Bacteriophage therapy	973	A. Sulakvelidze	2001	Antimicrob. Agents Ch
3	Dispersing biofilms with engineered enzymatic bacteriophage	516	T. K. Lu	2007	Proc. Nat. Acad. Sci. U.S.A
4	A controlled clinical trial of a therapeutic bacteriophage preparation in chronic otitis due to antibiotic-resistant *Pseudomonas aeruginosa*; a preliminary report of efficacy	493	A. Wright	2009	Clin. Otolaryngol.
5	Development and use of personalized bacteriophage-based therapeutic cocktails to treat a patient with a disseminated resistant *Acinetobacter baumannii* infection	469	R. T. Schooley	2017	Antimicrob Agents Ch
6	A bacteriolytic agent that detects and kills *Bacillus anthracis*	465	R. Schuch	2002	Nature
7	Phage cocktails and the future of phage therapy	434	B. K. Chan	2013	Future Microbiol.
8	Engineered bacteriophages for treatment of a patient with a disseminated drug-resistant *Mycobacterium abscessus*	429	R. M. Dedrick	2019	Nat. Med.
9	The innate immune modulators staphylococcal complement inhibitor and chemotaxis inhibitory protein of *Staphylococcus aureus* are located on beta-hemolysin-converting bacteriophages	400	Willem J. B. van Wamel	2006	J. Bacteriol.
10	Emerging strategies to Combat ESKAPE pathogens in the era of antimicrobial resistance: A review	398	M. S. Mulani	2019	Front. Microbiol.
11	Phage therapy in clinical practice: treatment of human infections	397	E. Kutter	2010	Curr. Pharm. Biotechnol.
12	Human volunteers receiving *Escherichia coli* phage T4 orally: a safety test of phage therapy	367	A. Bruttin	2005	Antimicrob Agents Ch
13	Rapid killing of *Streptococcus pneumoniae* with a bacteriophage cell wall hydrolase	354	J. M. Loeffler	2001	Science
14	Bacteriophage endolysins--current state of research and applications	348	M. J. Loessner	2005	Curr. Opin. Microbiol.
15	Bacteriophage therapy rescues mice bacteremic from a clinical isolate of vancomycin-resistant *Enterococcus faecium*	342	B. Biswas	2002	Infect Immun.

### Keywords analysis of research hotspots

**Figure 8:**
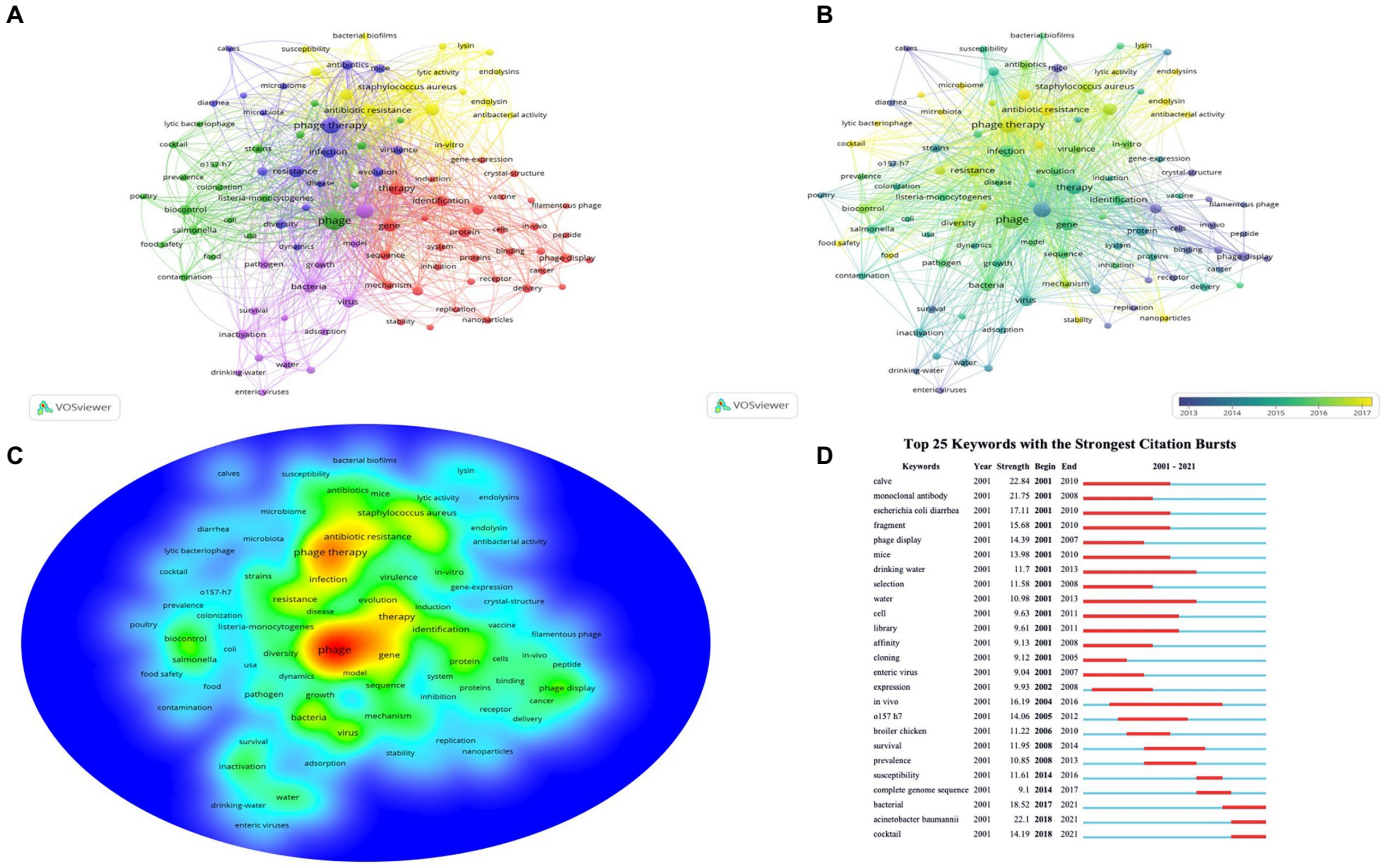
The visualization map of keywords. **(A)** Network visualization of keywords based on VOS viewer. **(B)** The overlay visualization map of keywords based on VOS viewer. **(C)** Density visualization map of keyword co-occurrence based on VOS viewer. **(D)** The top 25 keywords with the strongest citation bursts from 2001 to 2021 based on Cite Space. The red bars represent the strongest citation bursts time period.

Based on keyword analysis, we can identify past research hotspots and predict development trends. Among 6,538 publications, a total of 20,571 keywords were identified in our analysis. The minimum number of keyword occurrences was set to 75, obtaining the top 100 keywords (Figures 8A–C). As shown in Figure 8A, all keywords can be clustered into five categories: cluster #1 “genetic characteristics and mechanism of bacteriophage” (color in red) contained 34 keywords, cluster #2 “phages research in *Salmonella* or *Listeria* and epidemiological studies” (color in green) contained 21 keywords, cluster 3 “evolution and diversity of phages and research on the microbiome” (color in blue) contained 17 keywords, cluster 4 “multi-drug bacteria and biofilms” (color in yellow) contained 16 keywords, and cluster 5 “phage microbial characteristics” (color in purple) contained 16 keywords. Moreover, the top 10 most frequent keywords in order of occurrence were: “bacteriophage” (1,589 times), “phage therapy” (1,174 times), “*Escherichia-coli”* (842 times), “infection” (553 times), “resistance” (548 times), “bacteriophage therapy” (444 times), “*Pseudomonas aeruginosa”* (406 times), “*in vitro*” (359 times), “*Staphylococcus aureus*” (348 times), and “protein” (329 times). Figures 8B,C show that “biofilm, ““lysin, ““endolysin, ““nanoparticles, ““host-range, “and “antibiotic resistance” are the hot topics in this field of research right now.

Figure 8D lists the top 25 keywords with the strongest citation burst from 2001 to 2021. The top five strongest burst keywords were “calve” (2001–2010, strength = 22.84), “monoclonal antibody” (2001–2008, strength = 21.75), “*Escherichia coli”* (2001–2010, strength = 17.11), “fragment” (2001–2011, strength = 15.68), “phage display” (2001–2008, strength = 14.39), respectively. Most of the keyword bursts were generated between 2001 and 2010, while “complete genome sequence” (2014–2017, strength = 9.1) and “susceptibility” (2014–2016, strength = 11.61) appeared after 2014. Besides, the bursts of keywords “bacterial, ““cocktail, “and “*Acinetobacter baumannii*” were still ongoing.

## Discussion

Bacterial antibiotic resistance has become a severe problem in recent years, and phage therapy offers renewed hope to combat multi-drug resistant bacteria (Lin et al., 2017; Azimi et al., 2019; Suh et al., 2022). However, the past research emphasis in the field of PT for bacterial infections and the hot spots and future research trends in recent years are still not thoroughly investigated. Bibliometrics is an emerging tool for quantitatively analyzing literature (Wang et al., 2020). Therefore, we used the WoSCC database and performed a comprehensive bibliometric visualization analysis in this field. Our study selected 6,538 papers with a total of 227,633 citations, and all authors were from 119 countries or regions for all publications. In terms of the number of publications, the annual number of publications has increased significantly from 2001 (98 publications) to 2021 (976 publications), and the number of publications in the last 5 years has increased rapidly (Figure 2A). This may suggest that more scientists are interested in PT research, and the current upward trend implies that more papers will be published on this subject in the future.

Regarding regional distribution, Europe, North America, and East Asia are the regions with the largest number of publications in this field. The analysis of the research productivity of countries not only demonstrates their rank in the field but also helps to judge a country’s science policy and thereby helps to adjust their science funding (Fiala, 2012; Deng et al., 2020). Figure 3 shows that the United States has been the most productive country in PT research since the 21st century, and the United States is far ahead of the other countries. Table 1 shows that the United States, China, and the United Kingdom were the top three productive countries. Moreover, the percentage of total publications from the United States (28.56%) is approximately equal to the sum of the remaining four countries in the top five, namely China (12.41%), the United Kingdom (7.08%), Germany (6.23%), and Canada (5.83%). From the citation network map, we can also see that the United States has the largest TLS, which suggests that publications from the United States have a higher impact. The United States’s advantage is also seen in the funding agencies, with half of the top 10 funding agencies coming from the United States (Table 3). As the most productive country, the United States has the overwhelmingly highest number of publications, total citations, and H-index, indicating that the United States might maintain the dominant position. However, this gap with the United States has narrowed in recent years as the number of publications in China has increased dramatically. Notably, all three countries from Asia, including China, India, and South Korea, started late but have become the main productive contributors in recent years. The current international cooperation map results showed that the United States, as a research center, has close cooperation with Canada, Australia, and China, as well as with European countries, especially the United Kingdom, Germany, and France (Figure 3A). China cooperates actively with South Korea, Japan, and the United States (Figure 3B).

As for affiliations, European countries account for seven of the top 10 most productive affiliations. The Polish Academy of Sciences is in the first place, followed by Ku Leuven University with an H-index of 45 and 46, respectively, and the Chinese Academy of Sciences is in third place with an H-index of 26 (Table 2). Harvard University ranked first with a 68.26 average citation rate, followed by Ghent University (47.19) and Ku Leuven University (49.39). The network map of institutional cooperation analysis indicated that the Polish Academy of Sciences had close collaborations with the University of Gdańsk; Harvard University had close collaborations with Texas A&M University and Monash University; and the Chinese Academy of Sciences had close collaborations with Shanghai Jiao Tong University, Jilin University, and the Jiangsu Academy of Agricultural Sciences.

The top 10 most productive journals were all high-quality journals with Q1 or Q2 in the JCR classification, and most of the journals had an IF between 5 and 10 (average IF = 6; Table 4). *Frontiers in Microbiology* (Q1, IF = 6.064), *Viruses Basel* (Q2, IF = 5.818), and *Applied and Environmental Microbiology* (Q2, IF = 5.005) were the journals that published the most topics related to phage treatment of bacterial infections. *Applied and Environmental Microbiology* is the most cited journal, followed by *Antimicrobial Agents and Chemotherapy* and *Frontiers in Microbiology*. Thus, articles relevant to the field are more likely to be published in the above journals. The visual analysis of journals with a co-citation network map reveals the information flow between journals. In this study, *Frontiers in Microbiology* and *Viruses Basel* showed the highest centrality (Figure 5). Four of the top 10 prolific authors were from Poland; two were from Spain, and the others were from Belgium, Portugal, the United States, and Australia (Table 5). A. Gorski and B. Weber-Dabrowska from the Polish Academy of Sciences ranked first and third, respectively. Lavigne R. from Belgium has the second-highest number of publications with the highest H-index. However, regarding the number of citations, V. A. Fischetti has the total height of citations with 4,315 times. Co-authorship analysis is a research method that measures the relationship between co-authored literature (Wu et al., 2021a). Co-authorship analysis revealed that A. Gorski, S. T. Abedon, A. Sulakvelidze, V.A. Fischetti, B. K. Chan, et al. were in the center of the cooperating clusters (Figure 6).

The top 15 most co-cited articles related to PT in bacterial infection studies are given in Table 6. A highly cited article is generally defined as high-quality research that is groundbreaking or has a significant impact in a field and is often highly recognized by peer researchers. Most of these highly cited articles are published in high-quality journals, including *Nature*, *Science*, *FEMS Microbiology Review*s, and *The Proceedings of the National Academy of Sciences* (*PNAS*). Twelve highly cited articles were published before 2010, and three studies were published after 2010. The most highly cited article, written by Weinbauer (2004), provides a very detailed description of the ecological characteristics of phages. It provides a good knowledge framework for subsequent phage-related studies (Weinbauer, 2004). The second most cited publication is a mini-review published by Sulakvelidze et al. (2001). The authors express their concerns about antimicrobial drug-resistant pathogenic bacteria, share their perspectives on the future clinical application of PT research, review in detail the PT-related studies conducted in Poland and the former Soviet Union, and analyze the reasons why the clinical application of PT has failed to develop in the West (Sulakvelidze et al., 2001). The third most cited paper published in *PNAS* by Lu and Collins (2007), through a synthetic biology technique, phages were engineered to express a biofilm-degrading enzyme during infection to attack both the bacterial cells in the biofilm and the biofilm matrix consisting of extracellular polymers. The engineered phage greatly improves the efficacy of biofilm elimination, which brings a new therapeutic option to bacterial biofilms that are difficult to eliminate by traditional antibacterial drugs. All three highly cited articles published after 2010 focused on multiple drug-resistant (MDR) bacteria, while engineered bacteriophages or personalized bacteriophage-based therapeutic cocktails for MDR bacterial infections have shown favorable results (Schooley et al., 2017; Dedrick et al., 2019; Mulani et al., 2019). It also suggests that we are confronted with the severe issue of antibiotic-resistant bacterial infections, for which PT would be a good study topic.

Co-cited reference burst analysis and timeline view of 11 clusters of co-cited references were performed by CiteSpace software. Compared to the early burst of citations, five articles published later than 2015 continue to be cited and may be more highly cited over time. Nobrega et al. (2015) published a review in *Cell*, and the authors present the main obstacles encountered in PT and the solutions to circumvent them, mainly focusing on the genetic modification of phages to obtain desirable biological properties to overcome the limitations of phages as therapeutic agents. Pires et al. (2016) not only highlighted the great potential of phages as antimicrobial agents but also focused more on other applications of phages, i.e., the future application of phages being developed as vehicles for drug delivery or vaccines and the assembly of new materials. Chan et al. (2016) discovered OMKO1 phages as a new approach to PT, where phages play a selective role in making multidrug-resistant bacteria increasingly sensitive to conventional antibiotics. This approach using phages as targeted antibiotics could extend the lifespan of current antibiotics and potentially reduce the incidence of antibiotic-resistant infections (Chan et al., 2016). Sarker et al. (2016) treated children with acute bacterial diarrhea with PT; although there were unable to achieve improved diarrheal outcomes, oral phage showed safe intestinal transport in children. A timeline view of clusters of co-cited references showed that #0 (phage therapy), #2 (*Pseudomonas aeruginosa* infection), and #7 (binding domain) were hot research topics in recent years.

The keyword analysis reveals the changing trends of research hotspots in academic fields as an essential part of bibliometric analysis (Ma et al., 2022). As shown in the keyword co-occurrence analysis by the VOS viewer, a total of 20,571 keywords were extracted from the 6,538 papers. The minimum number of keyword occurrences was set to 75, obtaining the top 100 keywords, bacteriophage, phage therapy, *Escherichia-coli*, infection, resistance, bacteriophage therapy, *Pseudomonas-aeruginosa*, *in vitro*, *Staphylococcus aureus*, and protein were the most frequently occurring keyword. In Figures 8A–C, those five clusters based on the timeline can be divided into three phases: cluster #1 earlier phage research focused mainly on the exploration of the biology and genomic features of phages such as gene (gene-expression), protein, mechanism, receptors, sequence, inhibition, phage display, delivery, and replication. Furthermore, clusters #2 and #5 were more popular research topics in the field around 2015, with high occurrence keywords such as *Escherichia coli*, *Salmonella*, biocontrol, inactivation, adsorption, strains, colonization, growth, pathogen, and phage epidemiology. Then the current research focus is mainly on cluster #3 and cluster #4, including *in vitro* experiments, infectious diseases caused by MDR, cocktail therapies, bacterial biofilms, lysins, lytic activity, endolysin, and the microbiome. Besides, topics such as food safety, nanoparticles, and stability are likely to attract more attention in the future.

Keywords with the strongest citation burst can reflect the attention of researchers over a certain period of time, and by observing the trend of keywords over time, the future trend can be reasonably predicted (Wu et al., 2021b,c). Figure 8D lists the top 25 keywords with the strongest citation burst from 2001 to 2021, which analysis by CiteSpace. Keywords, such as calve, monoclonal antibodies, phage display, and fragment began a high burst of keywords from 2001 to 2011. Monoclonal antibodies (mAbs) isolated through phage display technology, multiple recombinant antibodies against various antigens can be obtained in a short period through phage display technology, and some of the mAbs isolated through phage display technology have been approved for the treatment of cancer, autoimmune diseases, cardiovascular diseases, and infectious diseases with great success (Liu, 2014; Frenzel et al., 2016; Kumar et al., 2019; Alfaleh et al., 2020). The wide use of complete genome sequencing technologies also became a hot topic in PT studies around 2014. Applying complete genome sequencing technology, we can obtain bioinformatics of phage biology and complete genomes and fully understand different phage characterizations to obtain critical evidence in PT (Hatfull, 2020; Wang et al., 2021). Recent PT studies have increasingly focused on the effects and safety of their use in animals or humans. Several commercial phage products approved by the FDA are available in the food industry (Lin et al., 2017). These agents are effective against *Listeria monocytogenes*, MRSA, *Escherichia coli* O157: H7, *Salmonella*, *Mycobacterium tuberculosis*, *Campylobacter*, and *Pseudomonas syringae* (Monk et al., 2010; Schofield et al., 2013; Endersen et al., 2014). The development of PT as an adjunct to conventional antibiotics has gained substantial momentum. One study reviewed PT research from 2008 to 2021, including 20 animal studies, 35 clinical case reports or case series, and 14 clinical trials, in which PT demonstrated relative safety as well as efficacy in animal studies, and Phage has shown great potential in the adjuvant treatment of cystic fibrosis worsening, bone/joint infections, pneumonia, bacteremia, urinary tract infections (UTI), endocarditis, and cardiothoracic surgery-associated infections, suggesting that more and more researchers or clinicians are considering PT in the treatment of MDR bacterial diseases (Liu et al., 2021). Salmond and Fineran (2015) introduced in great detail the influence of phage research on biology in the past 100 years, and it is not difficult to find that bacteriophage-related research has entered a stage of rapid development since the 21st century. Schooley et al. (2017) reported promising outcomes using personalized bacteriophage-based therapeutic cocktails for the treatment of MDR *A*. *baumannii* infection in 2017, and this study has attracted widespread attention. At the same time, our results showed that the still ongoing bursts of keywords were bacterial, cocktail, and *Acinetobacter baumannii*, which were completely consistent with the current research hotspots and trends in the field. Also, our data showed that phage research hotspots gradually shift from basic research to clinical applications based on the keywords with the strongest citation burst analysis.

## Limitations

Our work has a few limitations that need to be addressed. Firstly, this study followed the standard practice of bibliometric analyses by restricting its search to English-language articles and reviews in a single WoSCC database. It resulted in excluding other databases and important works written in languages other than English. Moreover, keyword search strategies inherently introduce bias in bibliometric analyses. However, we believe this will not adversely affect the overall trend analysis because the WoSCC database is the most commonly used in bibliometric analyses, and we have carefully selected relevant search terms. The primary research trends might be reflected in these English publications. Second, as reported by other bibliometric studies, different analysis software and tools’ various algorithms may result in a particular bias (Wu et al., 2021a; Zhang et al., 2022). Lastly, we regret that we were unable to discuss and cite several excellent articles in this field due to space constraints.

## Conclusion

Phage therapy for bacterial infections was investigated bibliometrically in the current study over the period of 2001 to 2021. It has been discovered that researchers all over the world are starting to pay more attention to this subject. The most prominent provider is the United States, but China has progressively risen to prominence as a significant participant in recent years. *Frontiers in Microbiology* and *Viruses Basel* were the most productive journals for publishing new studies in this field. Phage therapy is progressively beginning to be applied from basic to clinical research. Nevertheless, additional clinical trials are required to investigate the potential value of phages in the post-antibiotic era while simultaneously ensuring their safety.

## Data availability statement

The original contributions presented in the study are included in the article/supplementary material, further inquiries can be directed to the corresponding authors.

## Author contributions

ZM designed the study. ZM and ZL were responsible for data collection. ZM, ZL, CX, WC, and JC analyzed the data and drafted the manuscript. WC and JC revised and approved the final version of the manuscript. All authors contributed to the article and approved the submitted version.

## Funding

This work was supported by the National Natural Science Foundation of China (Grant Number 82002323) and the National Key R&D Program of China (Grant Number 2020YFC2004900).

## Conflict of interest

The authors declare that the research was conducted in the absence of any commercial or financial relationships that could be construed as a potential conflict of interest.

## Publisher’s note

All claims expressed in this article are solely those of the authors and do not necessarily represent those of their affiliated organizations, or those of the publisher, the editors and the reviewers. Any product that may be evaluated in this article, or claim that may be made by its manufacturer, is not guaranteed or endorsed by the publisher.

## Footnotes

^1^ https://bibliometric.com
